# Rare Coronary Anomaly of Posterior Descending Artery Arising from Superdominant Left Anterior Descending Artery

**DOI:** 10.14797/mdcvj.1310

**Published:** 2023-12-29

**Authors:** Kanhai Lalani, M. Sudhakar Rao, R. Padmakumar, Pankti Parikh

**Affiliations:** 1 Kasturba Medical College, Manipal, Manipal Academy of Higher Education, Manipal, Karnataka, India; 2 St. John’s Medical College, St. John’s National Academy of Health Sciences, Bangalore, Karnataka, India

**Keywords:** congenital coronary anomalies, super-dominant left anterior descending artery, hyper-dominant left anterior descending artery

## Abstract

Coronary artery anomalies are uncommon anatomical variations that are usually detected incidentally during a coronary angiogram or computed tomography angiography. We report a case of a diabetic and hypertensive middle-aged male who presented with chest discomfort. Coronary angiography revealed no signs of coronary artery disease but showed a left anterior descending artery (LAD) looping around the left ventricular apex and running through the posterior interventricular groove as a posterior descending artery (PDA) beyond the crux. The nondominant right coronary artery (RCA) and left circumflex artery (LCX) had no connection with the PDA. The patient’s diabetic and hypertensive medications were adjusted, and he remained asymptomatic after 3 months. Interventionalists should be aware of the types of coronary anomalies that may complicate diagnosis and management during percutaneous coronary intervention.

## Description

Congenital coronary abnormalities are uncommon anatomical variations that affect 1% to 2% of the population. As in our case, the majority of them are detected by chance during a coronary angiogram (CA) or computed tomography angiography (CTA).^1^

A diabetic and hypertensive middle-aged gentleman presented with non-anginal chest discomfort that had persisted for 2 days. An electrocardiogram (ECG) revealed modest left ventricular hypertrophy (LVH), and an echocardiogram revealed concentric LVH. Serum troponin levels were within the usual range. At 10 metabolic equivalents (METS), the treadmill test revealed inducible ischemia.

**Figure 1:**
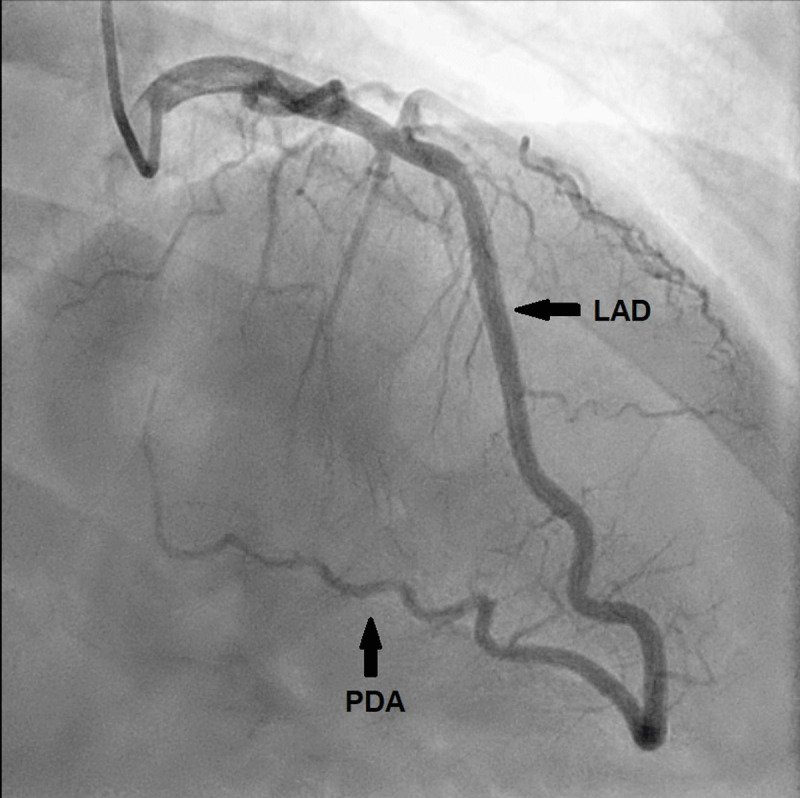
Coronary angiogram in right anterior oblique with cranial angulation showing the left anterior descending (LAD) artery wrapping around the apex and continuing as the posterior descending artery (PDA).

**Figure 2:**
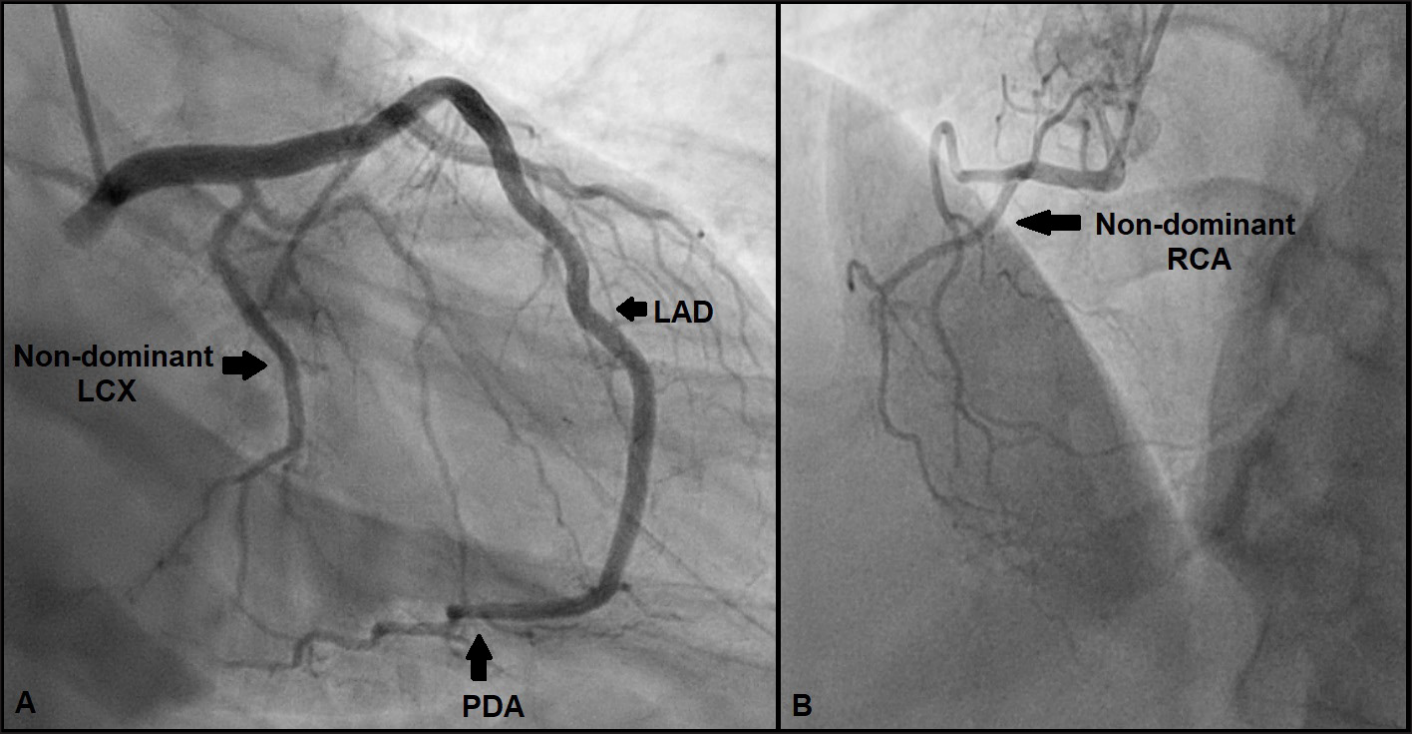
**(A)** Right anterior oblique with caudal angulation view; left anterior descending (LAD) artery wrapped around apex and continued as posterior descending artery (PDA). Nondominant left circumflex artery (LCX) is seen. **(B)** Left anterior oblique with cranial angulation view; nondominant right coronary artery (RCA) is seen.

Coronary angiography revealed no signs of coronary artery disease but showed a left anterior descending artery (LAD) looping around the left ventricular apex and running through the posterior interventricular groove as a posterior descending artery (PDA) beyond the crux (Figure 1, Video 1). The nondominant right coronary artery (RCA) and left circumflex artery (LCX), which supplied a lesser area of heart, had no connection with the PDA (Figure 2, Video 2). His diabetic and hypertensive medicines were adjusted, and he was asymptomatic 3 months later.

**Video 1:** Coronary angiogram in right anterior oblique with cranial angulation showing the left anterior descending artery wrapping around the apex and continuing as the posterior descending artery. See also at https://youtube.com/shorts/oYxoP4QGoX4.

**Video 2:** Right anterior oblique with caudal angulation view; left anterior descending artery wrapped around apex and continued as posterior descending artery. Nondominant left circumflex artery is seen. See also at https://youtube.com/shorts/zNNuF2aqDIM.

The origin of the PDA from the RCA or LCX defines coronary circulation dominance. The LAD, which continues as a PDA, is referred to as “hyperdominant LAD” or “superdominant LAD.” It is an exceedingly uncommon coronary abnormality, with just a few cases recorded in the literature. It can be an incidental observation during a CA, but its presence in the context of an acute coronary syndrome (ACS) leads to a bigger anterior and inferior myocardial infarction. Interventionalists must be familiar with the types of coronary anomalies that might complicate diagnosis and management during percutaneous coronary intervention.^2,3^

## Take-home Messages

- A rare coronary anomaly in which the left anterior descending artery (LAD) continues as a posterior descending artery is known as “hyperdominant LAD.”
- Acute coronary syndrome in the hyperdominant LAD territory can be catastrophic because it involves a greater infarction of the left and/or right ventricular myocardium.
- With a high index of clinical suspicion, early detection and treatment are crucial.
